# Tidal effects on periodical variations in the occurrence of singing humpback whales in coastal waters of Chichijima Island, Ogasawara, Japan

**DOI:** 10.1038/s41598-022-24162-0

**Published:** 2022-11-16

**Authors:** Koki Tsujii, Tomonari Akamatsu, Ryosuke Okamoto, Kyoichi Mori, Yoko Mitani

**Affiliations:** 1 Ogasawara Whale Watching Association, Aza Higashimachi, Chichijima, Ogasawara-Mura, Tokyo, 100-2101 Japan; 2 Graduate School of Environmental Science, Hokkaido University, 20-5 Benten-Cho, Hakodate, Hokkaido 040-0051 Japan; 3 Ocean Policy Research Institute, The Sasakawa Peace Foundation, 1-15-16 Toranomon, Minato-Ku, Tokyo, 105-8524 Japan; 4 Tokyo University of Marine Science and Technology, 4-5-7 Konan, Minato-Ku, Tokyo, 108-8477 Japan; 5 Teikyo University of Science, 2525 Yatsusawa, Uenohara, Yamanashi, 409-0193 Japan; 6 Wildlife Research Center, Kyoto University, 2-24 Tanaka-Sekiden-Cho, Sakyo, Kyoto, 606-8203 Japan

**Keywords:** Behavioural ecology, Marine biology

## Abstract

Marine organisms inhabiting coastal waters are known to be driven by periodic cycles such as diel, tidal, and seasonal changes. Humpback whales (*Megaptera novaeangliae*) breed in shallow and warm coastal waters, with males singing complex songs during the breeding season. To investigate periodic variations in humpback whale singing activities, we conducted fixed passive acoustic monitoring in the Ogasawara (Bonin) Islands, Japan, from winter to spring during 2016–2018. The singing activity and individual number of singers were observed throughout the day and night using a very long baseline passive acoustic array. The occurrence of singers peaked before sunrise and in the evening and was reduced during the daytime. The frequency of song reception depended on the tidal phase. A generalised additive model demonstrated that the occurrence of singers increased during the flood tide and decreased during the ebb tide in the waters west of Chichijima Island. These results suggest that the singing behaviour of humpback whales in breeding areas is affected by the diel and tidal cycles. Male humpback whales may change their behaviour or singing location depending on the strength and direction of the tidal current, considering that the selection of a stable location is beneficial for singing whales.

## Introduction

The behaviour of animals has seasonal and diel cycles, and that of many marine organisms inhabiting coastal waters is known to be associated with the tidal cycle in addition to the time cycle. In many fish species, changes in distribution and spawning behaviours associated with the tidal cycle have been reported^1–3^. Regarding marine megafauna, behaviours such as movement, distribution, and foraging of some cetaceans, sirenians, and pinnipeds are also known to be associated with tides and currents^4–9^. The tide-related behaviours of marine megafauna are often linked to the movement and distribution of prey species. Although there are few reports of behaviours that are not related to prey organisms, it has been reported that the vocal behaviour of male harbour seals (*Phoca vitulina*) in the mating season varies with female distribution and density related to the tide cycle^10^. The density of humpback whales (*Megaptera novaeangliae*) in the breeding area has also been suggested to be influenced by the speed of the current^11^. Understanding the natural behavioural patterns of these animals is important for assessing human impacts on marine wildlife and for proper conservation and management.

Humpback whales, a species of baleen whales that are marine megafauna, breed in shallow coastal waters^12–15^. In the North Pacific, the coastal waters of Hawaii, Mexico, Central America, Japan, the Philippines, and the Mariana Archipelago are known for their breeding areas^16,17^. Singing is a unique behaviour of male humpback whales^18–20^. During their breeding season, male whales produce a “song”, which is a complex vocal session composed of multiple sounds named “units”; a singing individual is called a “singer”. The functions of songs are unclear to date; however, singing behaviour is considered to play an important role in their mating strategies^20,21^.

Singing behaviour has been reported to be associated with the diel cycle in several regions. Sound pressure levels received by passive acoustic monitoring were significantly higher at night than during the daytime in Hawaii^22^. Diel variations in singing activity with a peak in singing activity at night and a low in the afternoon were observed off the coast of northern Angola^23^. In Okinawa, Japan, singing activity increased significantly after sunset, peaked at 22:00 h, and declined between sunrise and sunset^24^. However, diel patterns were not consistent between the two locations in American Sanoma^25^. Diel patterns with a decrease at midday and an increase at midnight were observed in Rose Atoll, whereas no diel patterns were observed in Tutuila^25^. As noted above, although many studies have reported diel variations with a peak after sunset of humpback whales^22–24^, it is possible that differences in daily movement patterns, habitat use, and whale behaviour depending on the island cause diel variations in singing activity^25^.

Information on the relationship between humpback whale vocal behaviour and tides is limited. A previous study reported that the sound production behaviour of humpback whales in the feeding area was affected by tidal conditions. Barlow et al.^26^ suggested that the occurrence of humpback whale feeding calls was indirectly related to tidal activity. In connection with feeding behaviour, Chenoweth et al.^27^ suggested that current direction and tidal amplitude affected the habitat selection of whales in southeastern Alaska. The horizontal and vertical distributions of prey species could be affected by the water current. Few studies have examined the relationships between the singing behaviour of humpback whales and the tidal cycle in breeding areas. Sousa-Lima and Clark^28^ demonstrated that the singing activity of humpback whales might also be influenced by the moon phase associated with the tidal cycle. Cerchio et al.^29^ also reported a significant reduction in singers during brighter moon phases. However, their discussions mainly focused on the effects of anthropogenic noise rather than tidal effects. This study aimed to reveal the periodic patterns of the singing activity of humpback whales, especially focusing on the tidal cycle in the breeding area.

**Figure 1:**
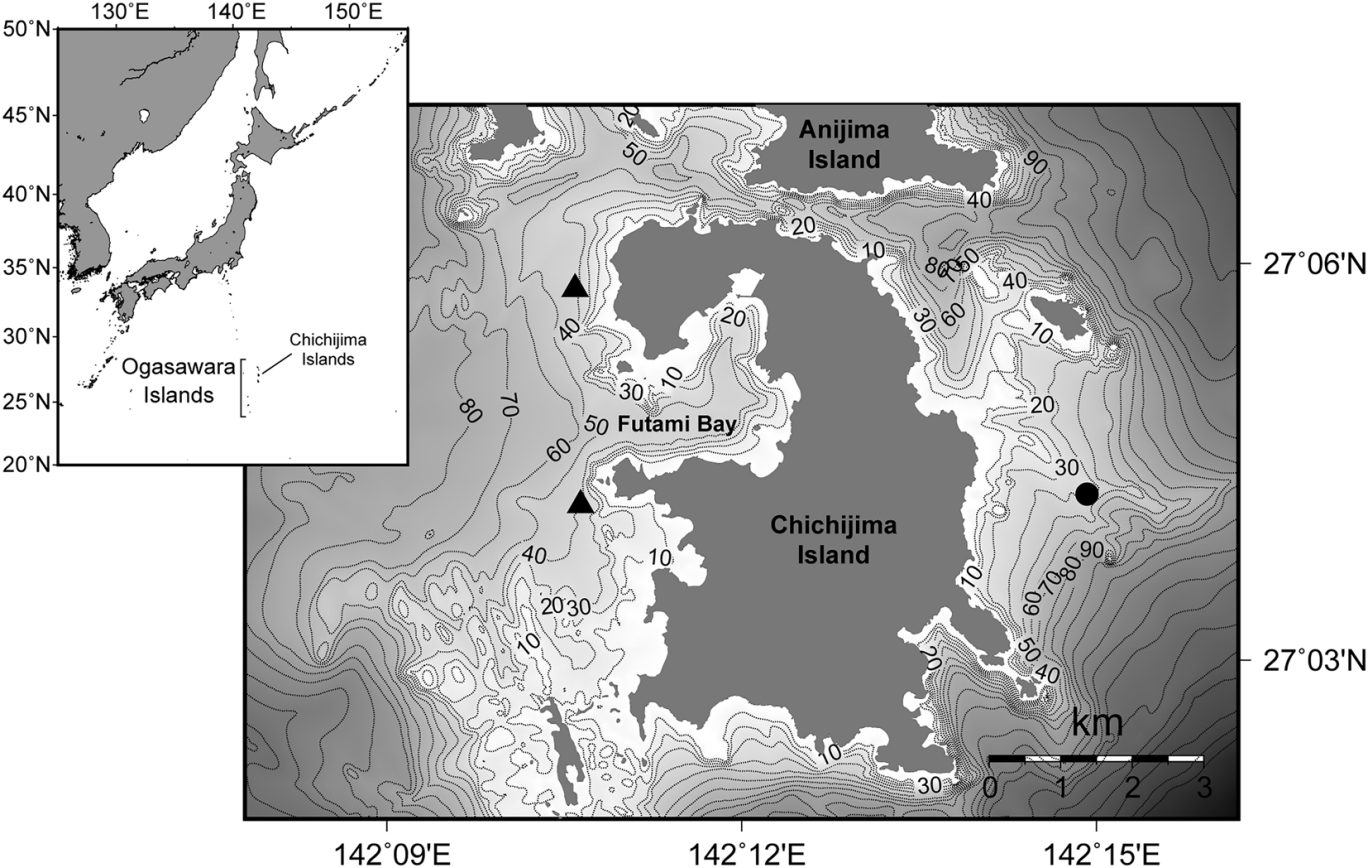
Maps of the study sites, Chichijima Island, Ogasawara, Japan. Black triangles (▲) and a black circle (●) indicate the mooring stations of the AUSOMS-mini stereo and the AUSOMS ver. 3.5, respectively. The bathymetry data was based on the M7023 digital bathymetry chart provided by the Japan Hydrographic Association. The figure was created using GMT (Generic Mapping Tools) version 5.4.3 (https://www.generic-mapping-tools.org/)^32^.

In the Ogasawara (Bonin) Islands of Japan, humpback whales of part of the western North Pacific stock gather for breeding from winter to spring^14,16,30,31^. In the present study, we conducted continuous passive acoustic monitoring using a very long baseline array in the near-shore waters of the Ogasawara Islands to observe periodic changes in the received song units and the number of singers in the breeding area of humpback whales (Fig. 1).

## Results

### Seasonality in humpback whale song occurrence

**Figure 2:**
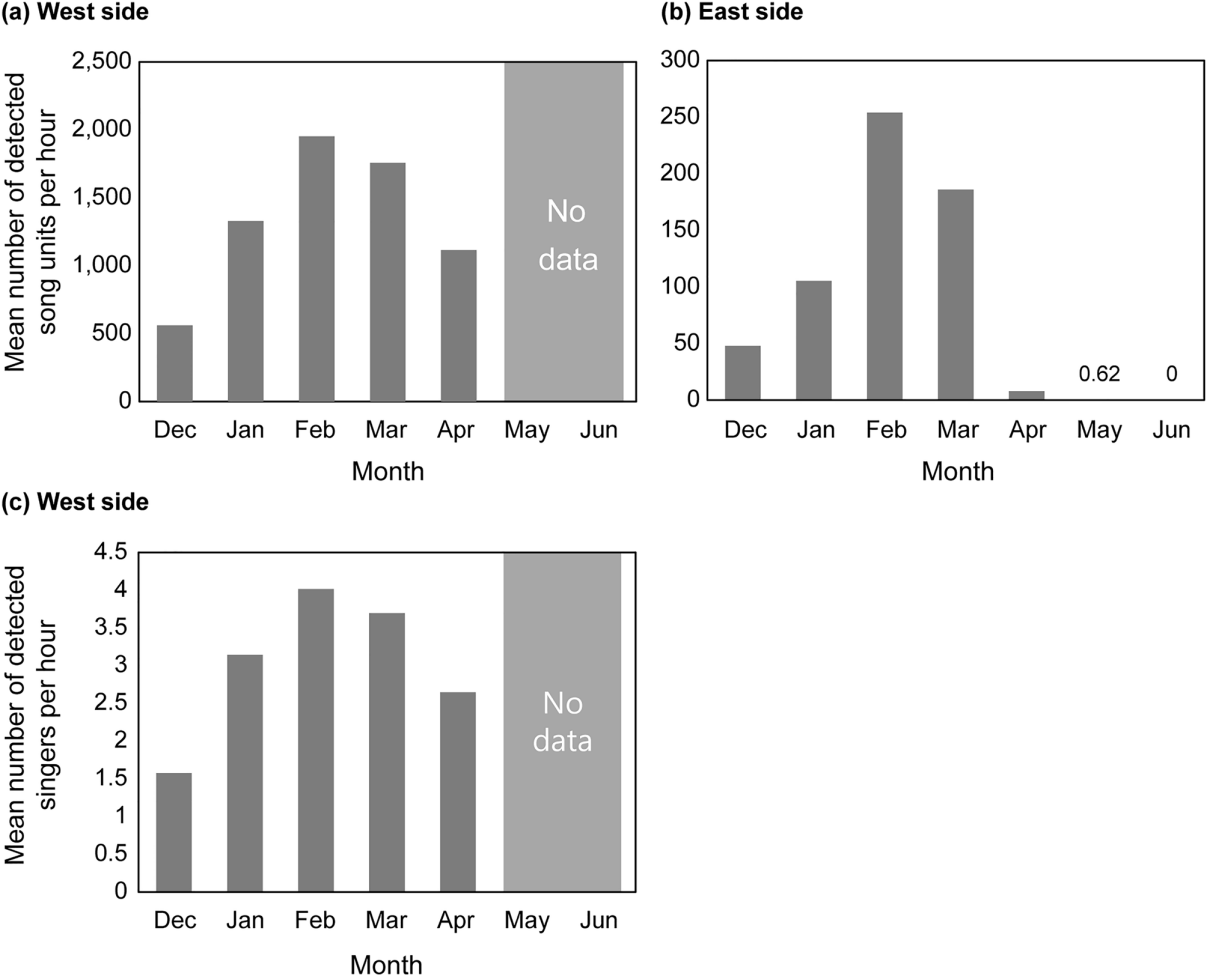
Seasonal variations in (**a**) the mean number of detected song units per hour on the west side of Chichijima Island; (**b**) the mean number of detected song units per hour on the east side of Chichijima Island; and (**c**) the mean number of detected singers per hour on the west side of Chichijima Island. The gray parts in (**a**) and (**c**) indicate that there is no data because recordings were not performed. Note that the y-axis scale is different in each graph.

Humpback whale songs were detected throughout the recording period (December–April) in the waters west of Chichijima Island (Figs. 2a,c). Song detections gradually increased from December to February, peaking in February and being greater during February–March than during other months. The number of song detections was lower in April than in January. In the waters east of the island, the number of song units detected per hour was approximately one-tenth less in all months than that in the west side (Figs. 2a,b). Songs were detected from December to May, but were not detected in June (Fig. 2b). Song detection increased steeply after January, occurred frequently during February–March and reached a peak in February, as with the west side. After March, song detections decreased considerably.

### Diel patterns in singing activity

**Figure 3:**
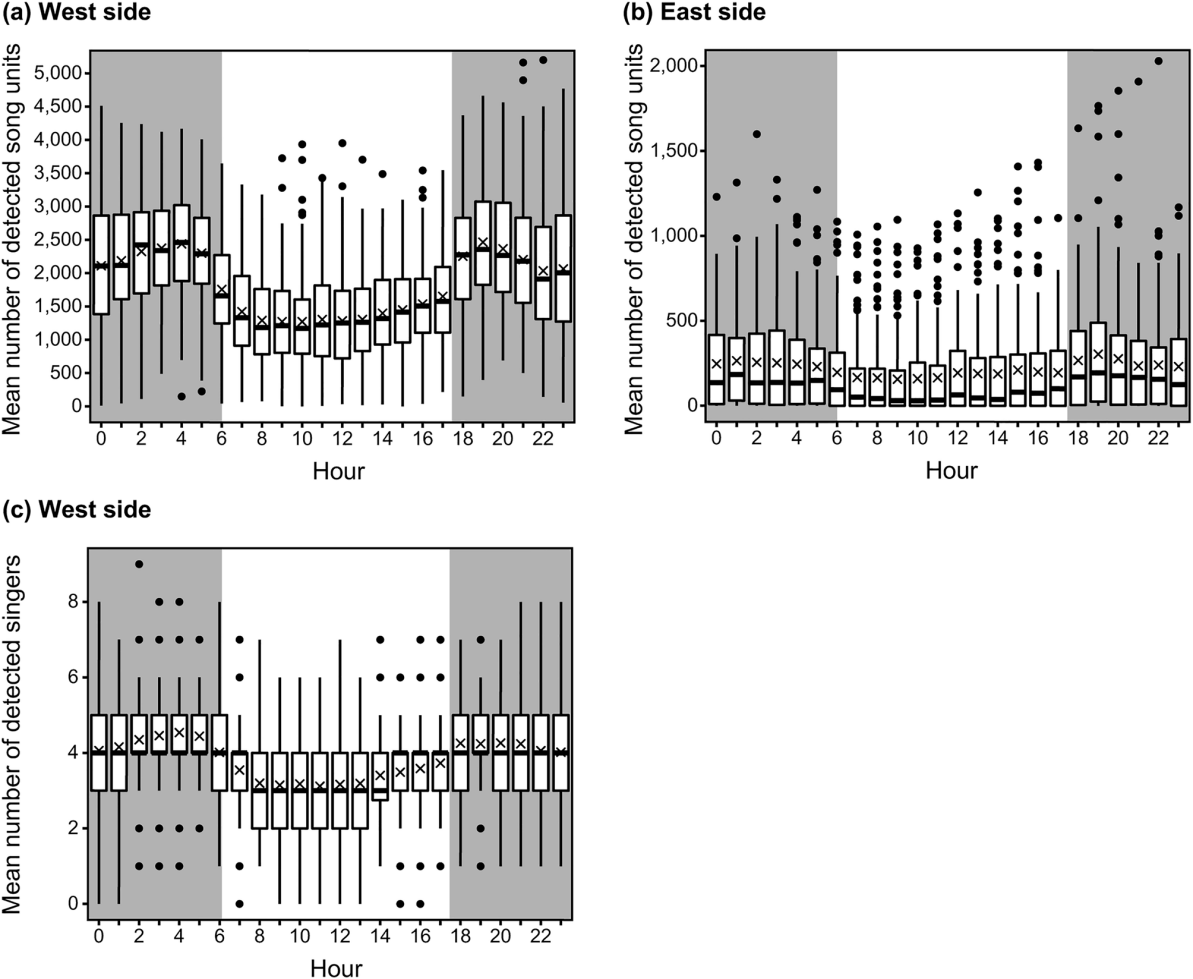
Diel variations in (**a**) the mean number of detected song units on the west side of Chichijima Island; (**b**) the mean number of detected song units on the east side of Chichijima Island; and (**c**) the mean number of detected singers on the west side of Chichijima Island. The lines in the middle of boxes represent the median, and the cross marks show the mean. The bottom and top of the boxes indicate the 25th and 75th percentiles. Whiskers represent 1.5 times the interquartile range, and dots outside of the whiskers represent outliers. Grey and white areas indicate night and daytime, respectively. Significant diel variations were observed on both sides of the island (Kruskal–Wallis rank sum test, all *p* < 0.001). Note that the y-axis scale is different in each graph.

The mean sunrise and sunset times between February and March were 05:54 ± 0:15 h (mean ± SD, n = 179) and 17:31 ± 0:09 h (mean ± SD, n = 179), respectively. During the peak occurrence season of humpback whale singers (February–March), song units and singers were observed throughout the day and night in the waters west and east of Chichijima Island. Significant diel variations in the mean number of detected song units were observed in the waters west and east of the islands (Kruskal–Wallis rank sum test, chi-squared = 877.22, *p* < 0.001 for the west side; chi-squared = 75.90, *p* < 0.001 for the east side, Figs. 3a,b). Significant diel variations in the mean number of detected singers were also observed in the waters west of the islands (Kruskal–Wallis rank sum test, Chi-squared = 449.75, *p* < 0.001, Fig. 3c). In the waters west of the island, the mean number of detected units and singers showed double peaks before sunrise (around 04:00 h) and after sunset (around 18:00 h and 19:00 h, respectively) (Figs. 3a,c). The values decreased from dawn to daytime, and increased from daytime to evening (Figs. 3a,c). The number of detected song units and singers at night was slightly lower than that in the evening and dawn (Figs. 3a,c). In the waters east of the island, many song units were detected from midnight to dawn, with a peak around 01:00 h, and in the evening, with a peak around 19:00 h (Fig. 3b). Song detection decreased from dawn to daytime and increased from daytime to evening (Fig. 3b). Although there was a slight difference in the peak hours, the fluctuations in the number of detected song units on the east side showed trends similar to those on the west side (Fig. 3b).

### Tidal effects on the occurrence of singing whales

**Figure 4:**
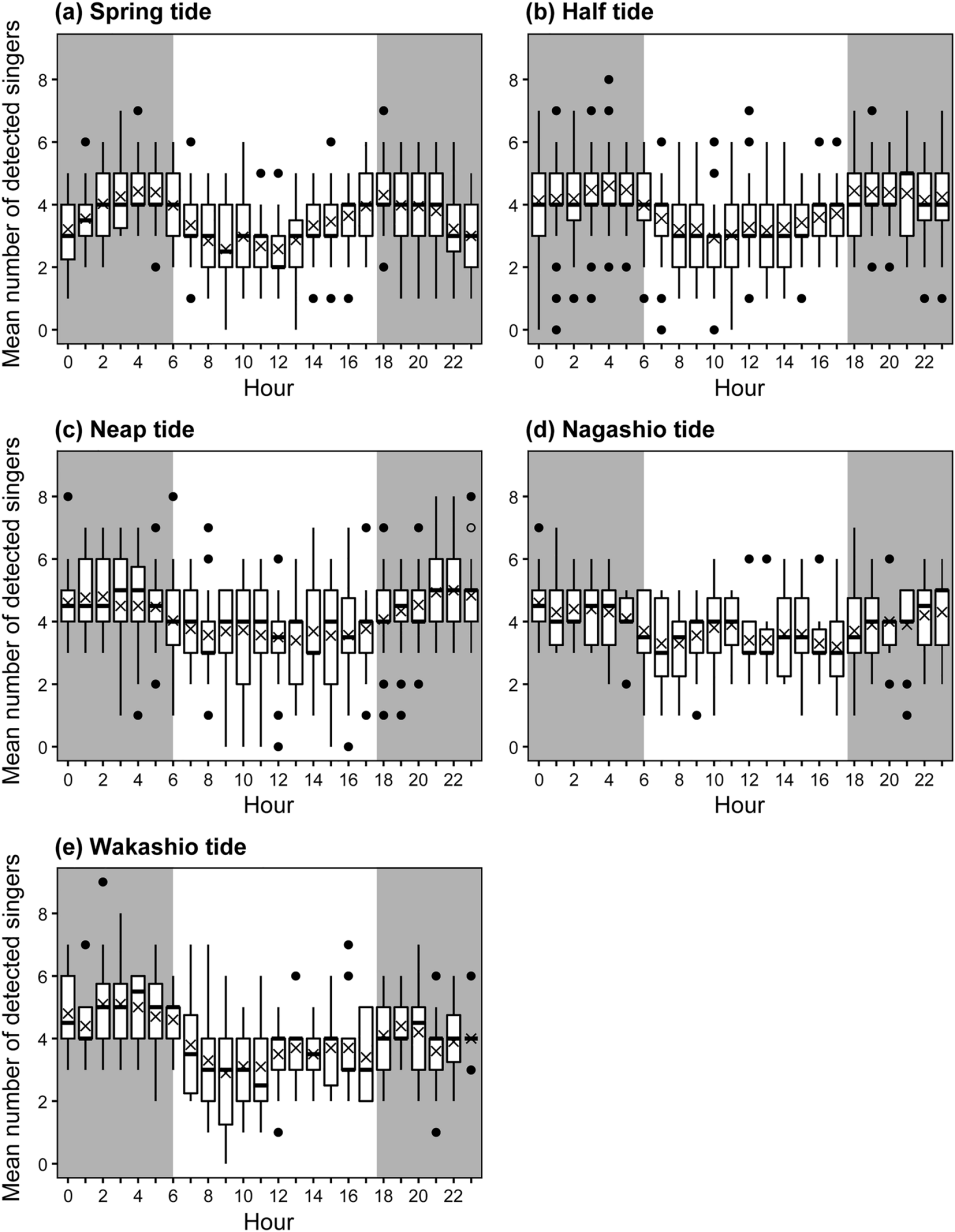
Diel variations in the mean number of detected singers for each tide type on the west side of Chichijima Island. The lines in the middle of boxes represent the median, and the cross marks show the mean. The bottom and top of the boxes indicate the 25th and 75th percentiles. Whiskers represent 1.5 times the interquartile range, and dots outside of the whiskers represent outliers. Grey and white areas indicate night and daytime, respectively.

The tidal conditions were classified into five types based on the moon age: spring, half, neap, Nagashio, and Wakashio tide (see Methods section). Spring tides have a large tidal range, neap tides have a small tidal range, and half tides are midway between the spring and neap tides. The Nagashio tide is a transitional tide from neap to half tide, and the Wakashio tide is a tidal condition on the day after the Nagashio tide. Of the 148 analysed days, the number of days for spring, half, neap, Nagashio, and Wakashio tides was 39, 59, 30, 10, and 10, respectively (Supplementary Fig. S1). The peak time and diel fluctuation patterns of the mean number of detected singers differed depending on tidal type (Fig. 4). During the spring tide, two distinct peaks were observed at 04:00 h and 18:00 h (Fig. 4a). The occurrence of singers at midnight was as low as that during the day. In the half-tide, two peaks were observed at 04:00 h and 18:00 h (Fig. 4b). In the neap tide, two peaks were observed at 02:00 h and 22:00 h (Fig. 4c). A single peak was observed at 00:00 h during the Nagashio tide (Fig. 4d), and two peaks were observed at 02:00 h and 22:00 h during the Wakashio tide (Fig. 4e).

**Figure 5:**
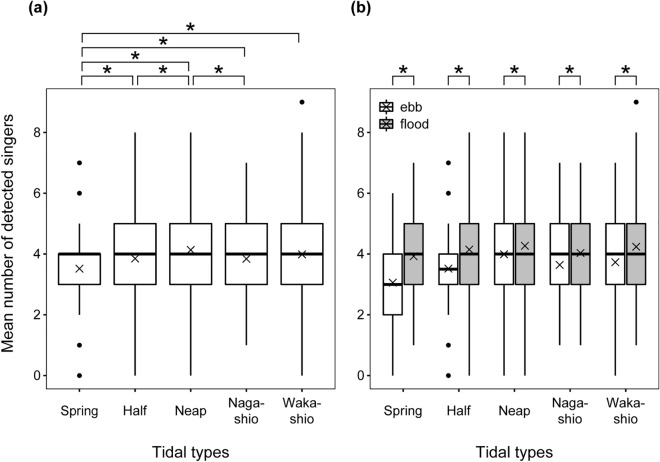
Differences in the mean number of detected singers: (**a**) for each tidal type and (**b**) between during the ebb and flood periods for each tidal type. The lines in the middle of boxes represent the median, and the cross marks show the mean. The bottom and top of the boxes indicate the 25th and 75th percentiles. Whiskers represent 1.5 times the interquartile range, and dots are outside of this range. Asterisks (*) indicate statistically significant differences between the groups (Steel–Dwass test, *p* < 0.05 in [**a**]; Mann–Whitney U test, *p* < 0.05 in [**b**]).

**Figure 6:**
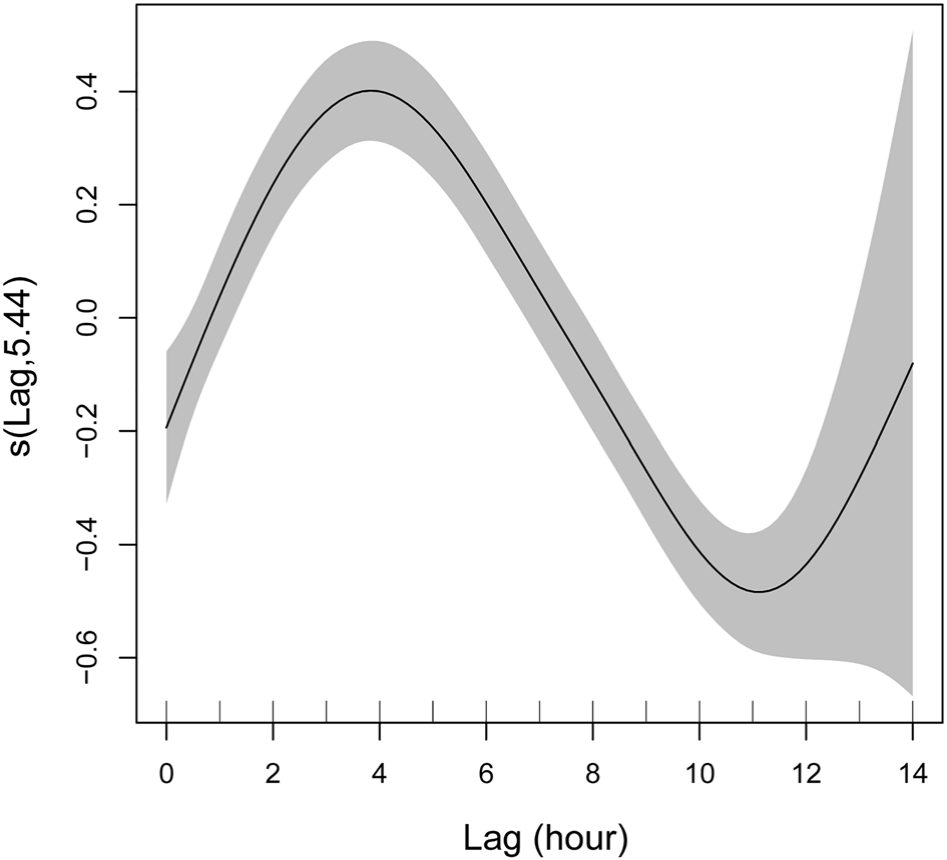
Relationships between the mean number of detected singers and the time lag from low tide on the west side of Chichijima Island. The x-axis shows the time lag from the low tide to the next low tide. The high tide time is 6 to 7 h after the low tide time. The scale of the smoothing effect is shown on the y-axis. A value greater than zero on the y-axis indicates a positive effect on the occurrence of singers, and a value less than zero indicates a negative effect on the occurrence of singers. The shaded area shows the 95th percentile confidence band around the smooth function.

The mean number of detected singers per hour for each tide type is shown in Fig. 5a. Singers were the least detected during spring tide (Steel–Dwass test, *p* < 0.001 for spring tide vs. half tide, spring tide vs. neap tide, and spring tide vs. Wakashio tide; *p* = 0.008 for spring tide vs. Nagashio tide, Table 1). In contrast, the most singers were detected in neap tide (Steel–Dwass test, *p* < 0.001 for neap tide vs. spring tide, neap tide vs. half tide; *p* = 0.02 for neap tide vs. Nagashio tide; *p* = 0.44 for neap tide vs. Wakashio tide, Table 1). In addition, significantly more singers were detected during flood periods than during ebb periods (Mann–Whitney U test, *p* < 0.001 for spring and half tides; *p* = 0.004 for neap tide; *p* = 0.036 for Nagashio tide; *p* = 0.025 for Wakashio tide, Table 1). The differences between the two tidal conditions were large during the spring and half tides (Fig. 5b, Table 1). The results of the generalised additive model (GAM) showed a significant effect of tidal shifts on the occurrence of singers (estimated degrees of freedom = 5.44, F = 28.49, *p* < 0.001, n = 3525, Fig. 6). Our model predicted that there was a positive effect on the number of singers during the period from low to high tide, and the effect reached a maximum around 4 h after low tide (Fig. 6). However, there was a negative effect during the transition from high to low tide (Fig. 6).

**Table 1:** Mean (± SD) of detected singers for each tidal type and between during the ebb and flood tide periods.

Tidal types	Mean ± SD (n)		
	All	Ebb	Flood
Spring tide	3.52 ± 1.25	3.06 ± 1.23	3.93 ± 1.12
	(n = 920)	(n = 433)	(n = 487)
Half tide	3.85 ± 1.29	3.52 ± 1.26	4.15 ± 1.24
	(n = 1411)	(n = 668)	(n = 743)
Neap tide	4.13 ± 1.42	3.99 ± 1.38	4.27 ± 1.46
	(n = 716)	(n = 349)	(n = 367)
Nagashio tide	3.84 ± 1.30	3.64 ± 1.32	4.03 ± 1.25
	(n = 239)	(n = 117)	(n = 122)
Wakashio tide	3.99 ± 1.51	3.73 ± 1.53	4.24 ± 1.45
	(n = 239)	(n = 118)	(n = 121)

## Discussion

Our results showed that the number of detected singing whales varied periodically depending on the diel, tidal (moon), and seasonal cycles.

A clear seasonal difference in song detection was observed in the study area. A high level of singing activity was observed from February to March, with a peak in February. This result was consistent with the migration peak revealed by sighting surveys in previous research^14^. More singing whales and song units were detected before sunrise and in the evening. This result is similar to the diel singing activity patterns observed in other breeding areas^22–25^. On Chichijima Island, a port exists only on the western side. Therefore, more vessel traffic was present on the west side than on the east side. However, the results showed similar diel patterns in the western and eastern waters, which implies that the influence of human activities on the diel patterns in the singing activities of humpback whales is limited. Additionally, nearly 10 times more song units were detected per hour on the west side than on the east side of the island (Figs. 2a, b). Although accurate comparisons were not possible because the recording systems were different, this result indicates that more singers use the west of the island than the east of the island. The waters west of Chichijima Island are considered a favourable environment for humpback whale singers.

Based on this, the reasons for the occurrence of such diel variations could be related to the behaviour and distribution of singers throughout the day. The behavioural hypothesis was that male humpback whales switch their mating strategies depending on the time of day. Because Ogasawara is the breeding area of humpback whales, they are thought not to forage here. Therefore, the effect of foraging behaviour, depending on prey species migration, on diel behaviour was excluded. In Hawaii, the mating behaviour of humpback whales was suggested to begin after sunrise, and male-male competition peaked in the afternoon^33^. In Okinawa, it was suggested that the number of singers might decrease because of an increase in the number of groups composed of multiple whales in the daytime^24^. Although the functions of singing behaviour are unclear, songs are considered to play the role of advertisements for females or other males^20,21,34^. As suggested by a previous study, male humpback whales may use acoustic signals more effectively during dark hours^22^. Our results are consistent with those of previous studies. The number of detected units and singers (Fig. 4) showed that male humpback whales were signalling during dim and dark hours, which may support the advertisement function of songs to females or other males.

Humpback whale singers can change their location depending on the time of the day. Munger et al.^25^ suggested that whales moved offshore or to the opposite side of the atoll during the day and closer to the recorder at night in American Samoa. However, in our study, the diel patterns of singing activity in the waters west and east of Chichijima Island were similar. It is unlikely to move back and forth between the two sides of the island. The diel variations in the number of singers could indicate that the humpback whales moved outside the detection range of the recorder (offshore, further south, or north) during the day and approached the shore near the recorders at dawn or dusk.

Interestingly, we found that the additional periodic occurrence of singers depends on the tidal type. A previous study also suggested that diel variations in humpback whale singing activity are influenced by the moon phase associated with tidal rhythms^28^. In our study, fewer singers were detected, and the diel variations were remarkable during the spring tide. However, the number of singers was the highest, and the diel variations were unclear during the neap tide. Therefore, we consider that the effect on singing activity is smaller when the tidal current is gentle. Weak current conditions may be suitable for whales to sing as whales typically sing in a stationary state underwater^35^. When the tidal current is fast, they are likely to drift away. If one of the functions of a song is to appeal to other whales, it is more acoustically effective to advertise at the same location without drifting. Therefore, male whales could choose other behaviours or move to other locations when the current is strong. This is considered an effective strategy from the perspective of cost-effectiveness.

The result of a GAM demonstrated that the occurrence of singers increased during the flood tide and decreased during the ebb tide in the waters west of Chichijima. In particular, during the spring tide, two distinct peaks occurred each day. A low tide with a large drop in tide level also occurred twice a day, around noon and midnight, during the spring tide (Supplementary Fig. S1). The decrease in the number of singers during the day and midnight at the spring tide was consistent with the timing when the tide was ebb. The ebb periods are not suitable for whales to sing, which could explain why two distinct peaks were observed during the spring tide. In the north and south of Chichijima, a strong tidal current flows from east to west during the flood tide and from west to east during the ebb tide^36^. In addition, the local people say that the tidal current flows from south to north and from north to south when the flood and ebb tides occur on the east and west coasts of Chichijima, respectively. The situation during flood tide might be beneficial for singers because they drifted northward, where there is no shadow area of sound transmission in deeper water areas. In contrast, drifting southward could reduce the communication distance because of shallow and complex bottom shapes (Fig. 1). Because the tide level drops during low tides, being close to shallow areas is even more disadvantageous for singers. Hence, on the western coast of Chichijima Island, the number of singers might have decreased because the current was strong and the direction of the flow was unsuitable for singing during the ebb tide in the spring tide.

This study has some limitations. We could not calculate the movement of individual singers during the day because of the limited bearing angle resolution of stereo passive acoustic monitoring. Acoustic observations in offshore areas will also be necessary to monitor the inshore-offshore movement of singers. Moreover, passive acoustic monitoring only detects phonating individuals, and no data will be accumulated for silent males, females, or calves. The results of the periodic behaviour shown in this study cannot be applied to all sexes and ages of whales.

In conclusion, the periodical variations in song units and the number of singing individuals of humpback whales in the Ogasawara waters were documented. At least three periodicities—seasonal, diel, and tidal cycles, contributed to the behaviour of the singers. Although further investigations focusing on changes in the behaviour and distribution of individual whales are needed, our results suggest that not only the diel period but also the tidal phase affects the behaviour of male humpback whales, including habitat selection. We hypothesised that a stable singing location could be beneficial for singers, which should be examined further. We propose a need for research that incorporates tidal effects to better understand the mating strategy of male whales and the role of songs.

## Methods

### Acoustic monitoring

Some of the datasets used in this study are the same as those used by Tsujii et al.^37^. Acoustic observations were conducted in the coastal waters of Chichijima Island, Ogasawara Islands, Japan, during the migrating season of humpback whales from 2016 to 2018. Autonomous underwater recorders were deployed at three locations (Fig. 1). Two recorders (AUSOMS-mini stereo; AquaSound Inc., Kobe, Japan) were deployed off the west side of Chichijima Island at a distance of approximately 3 km (27˚05′48.43″ N, 142˚10′35.48″ E and 27˚04′10.40″ N, 142˚10′38.30″ E) from February 2016 to March 2016, from February 2017 to April 2017, and from December 2017 to April 2018. The measurement frequency range of the AUSOMS-mini stereo is 100 Hz to 23 kHz. The range of the measured sound pressure levels was 70–160 dB re μPa. The AUOSMS-mini stereo can record continuously for up to approximately 21 days using two UM1 batteries and a 32-GB micro SD memory card. The recording period was constrained by the battery power, but not by the memory size. A compressed data format (MP3, 128 kbps) was used to reduce memory consumption, because we intended to perform continuous long-term recordings. This compressed format allowed for recording at a frequency of up to 17 kHz. In each deployment period, recorders were exchanged every two or three weeks before recording was stopped. Recorders were moored at 20 m, where the water depth was 40 m. Clocks of recorders that were deployed at two stations were synchronised at the beginning and end of the recording, which enabled measurement of the third bearing angle from the middle point of the two recorders. One recorder (AUSOMS ver. 3.5; AquaSound Inc., Kobe, Japan) was deployed off the east side of Chichijima Island (27˚04′15.41"N, 142˚14′55.20"E) from December 2016 to June 2017 and from January 2018 to April 2018 to compare diel variations on the opposite side of the island. AUSOMS ver. 3.5 had a hydrophone with a frequency response of 20 Hz to 20 kHz and a sensitivity of − 190 dB re 1 V μPa^−1^. The cut-off frequency of the low-pass filter was set to 20 kHz and the gain was set to 30 dB. We recorded using the WAV format with a sampling rate of 44.1 kHz and data resolution of 16 bits. The memory capacity of AUSOMS ver. 3.5 was 762 GB in total, comprising six 128-GB SDXC memory cards. AUOSMS ver. 3.5 can record continuously for up to approximately 100 days with two dedicated lithium batteries. In the first deployment period, the recorder was exchanged in March 2017 before the recording was stopped. The recorder was moored at 22 m, with a water depth of 48 m, along with the mooring system on the west side. Underwater sounds were continuously recorded at all locations. Permits and approvals for the deployment of recorders in Ogasawara waters were obtained from the Ogasawara Fisheries Cooperative Association.

### Detections of song units and separations of singing whales

The analyses included a total of 5,015 and 6,564 recorded hours on the west and east sides, respectively. Song units were automatically detected using custom-made software written in MATLAB (MathWorks, Natick, MA, USA)^37^. In the analysis of the data recorded on the west side of the island, each singer was acoustically distinguished using the recording data of the two recorders. Because the clocks of the two recorders were synchronised, the sound source directions of each song unit were obtained using the time difference arrivals of each unit detected from the two recorders. The direction changes in the song units that were detected continuously were visualised as dotted lines using Igor Pro (WaveMetrics Inc., Lake Oswego, OR, USA). The number of dotted lines per hour was manually counted as the number of singing whales. The number of song units per hour was then determined. To count the number of song units and singers, we used the sounds detected from both recorders. In the analysis of the data recorded on the east side of the island, we counted the number of song units per hour using one recorder. In acoustic processing, the sound pressure level of the raw data was raised by 30 dB using Adobe Audition CC (Adobe System Inc., San Jose, CA) before song detection because the recording level of the sound was low and the units were not detected well. The same detection parameters were used in all the acoustic analyses.

### Collection of sunrise–sunset time, moon age, and tidal data

The data on the times of sunset and sunrise and the age of the Moon were obtained from the National Astronomical Observatory of Japan (https://eco.mtk.nao.ac.jp/cgi-bin/koyomi/koyomix_en.cgi). The tidal conditions were categorised into five types based on the age of the moon: spring tide (Moon age = 0–2, 14–17, and 29), half tide (Moon age = 3–6, 12–13, 18–21, and 27–28), neap tide (Moon age = 7–9 and 22–24), Nagashio tide (Moon age = 10 and 25), and Wakashio tide (Moon age = 11 and 26).

Observational data on hourly tide levels on Chichijima Island (27˚06’N, 142˚12’E) were obtained from the Japan Meteorological Agency database (http://www.data.jma.go.jp/kaiyou/db/tide/genbo/genbo.php). The local minima and maxima were detected using the “islocalmin” and “islocalmax” commands of MATLAB from the time-series data of tide levels. The times when the tide values showed local minima and maxima were defined as low and high tide times, respectively. The tidal stages from low to high tides and high to low tides were defined as flood and ebb tides, respectively.

### Diel patterns in singing activity and tidal effects

We calculated the monthly fluctuations in the mean number of detected singers and units per hour using the results of the number of detected singers and units per hour. Ryan et al.^38^ described that it is appropriate to constrain the examination of diel variations at the time of the year when the songs are prevalent. Hence, in our analyses of diel patterns, only data from February to March, which had a high occurrence of singers, was used to consider the seasonal variations in the occurrence of singing whales and sampling effort bias. First, we calculated the mean number of detected singers or units by the time of day. The Kruskal–Wallis rank sum test was performed to determine whether there was a significant difference in the number of detected singers or units over time. Second, we calculated the mean number of detected singers by the time of day for the five tidal types using the data recorded on the west side to examine the tidal effects. We also compared the mean number of detected singers per hour among tidal types using the Steel–Dwass test. Then, we divided each tidal type into ebb and flood conditions and examined the differences in the number of detected singers between the ebb and flood periods using the Mann–Whitney U test. Finally, we constructed a GAM with a Gaussian distribution to predict the effects of tidal shifts on singing activity. The response variable was the mean number of detected singers per hour and the explanatory variable was the time lag from low tide to the next low tide. In the statistical analyses, the level of statistical significance was less than 5% (*P *< 0.05). All statistical analyses were performed using R, version 4.0.0^39^. In the GAM analysis, we used the “mgcv” package^40^.

## Supplementary Information

Supplementary Information.

## Data Availability

The datasets generated during and/or analysed during the current study are available from the corresponding author on reasonable request.
