# Author Correction: Tidal effects on periodical variations in the occurrence of singing humpback whales in coastal waters of Chichijima Island, Ogasawara, Japan

**DOI:** 10.1038/s41598-022-26874-9

**Published:** 2023-01-11

**Authors:** Koki Tsujii, Tomonari Akamatsu, Ryosuke Okamoto, Kyoichi Mori, Yoko Mitani

**Affiliations:** 1 Ogasawara Whale Watching Association, Aza Higashimachi, Chichijima, Ogasawara-Mura, Tokyo, 100-2101 Japan; 2 Graduate School of Environmental Science, Hokkaido University, 20-5 Benten-Cho, Hakodate, Hokkaido 040-0051 Japan; 3 Ocean Policy Research Institute, The Sasakawa Peace Foundation, 1-15-16 Toranomon, Minato-Ku, Tokyo, 105-8524 Japan; 4 Tokyo University of Marine Science and Technology, 4-5-7 Konan, Minato-Ku, Tokyo, 108-8477 Japan; 5 Teikyo University of Science, 2525 Yatsusawa, Uenohara, Yamanashi, 409-0193 Japan; 6 Wildlife Research Center, Kyoto University, 2-24 Tanaka-Sekiden-Cho, Sakyo, Kyoto, 606-8203 Japan

Correction to: *Scientific Reports* 10.1038/s41598-022-24162-0, published online 16 November 2022

The original version of this Article contained an error in the Results section, under the subheading ‘Tidal effect on the occurrence of singing whales’, where a citation to Figure 5, instead of a citation to Figure 6, was incorrectly inserted . As a result,

“Our model predicted that there was a positive effect on the number of singers during the period from low to high tide, and the effect reached a maximum around 4 h after low tide (Fig. 5).”

now reads:

“Our model predicted that there was a positive effect on the number of singers during the period from low to high tide, and the effect reached a maximum around 4 h after low tide (Fig. 6).”

The original Article has been corrected.

