# Supplementary material for: Tidal effects on periodical variations in the occurrence of singing humpback whales in coastal waters of Chichijima Island, Ogasawara, Japan
Source: Sci Rep. 2022 Nov 16;12:19702. doi: 10.1038/s41598-022-24162-0 (PMC9668835; doi:10.1038/s41598-022-24162-0)
Supplement: Supplementary file 1 — Supplementary Information. [file 41598_2022_24162_MOESM1_ESM.docx]

**Supplementary Information**

**Tidal effects on periodical variations in the occurrence of singing humpback whales in coastal waters of Chichijima Island, Ogasawara, Japan**

**Koki Tsujii ^1, 2, *^, Tomonari Akamatsu ^3^, Ryosuke Okamoto ^4^, Kyoichi Mori ^5^, and Yoko Mitani ^6^**

1 Ogasawara Whale Watching Association, Aza Higashimachi, Chichijima, Ogasawara-mura, Tokyo, 100-2101, Japan

2 Graduate School of Environmental Science, Hokkaido University, 20-5 Benten-cho, Hakodate, Hokkaido, 040-0051, Japan

3 Ocean Policy Research Institute, The Sasakawa Peace Foundation, 1-15-16 Toranomon, Minato-ku, Tokyo, 105-8524, Japan

4 Tokyo University of Marine Science and Technology, 4-5-7 Konan, Minato-ku, Tokyo, 108-8477, Japan

5 Teikyo University of Science, 2525 Yatsusawa, Uenohara, Yamanashi, 409-0193, Japan

6 Wildlife Research Center, Kyoto University, 2-24 Tanaka-Sekiden-cho, Sakyo, Kyoto, 606-8203, Japan

* Corresponding author: tsujii@owa1989.com





**Figure S1.** Diel variations in the mean tide level for each tide type in Chichijima Island (27˚06’N, 142˚12’E).
